# Spatial regulation of substrate adhesion directs fibroblast morphotype and phenotype

**DOI:** 10.1093/pnasnexus/pgae289

**Published:** 2024-07-25

**Authors:** Mirko D’Urso, Ignasi Jorba, Atze van der Pol, Carlijn V C Bouten, Nicholas A Kurniawan

**Affiliations:** Department of Biomedical Engineering, Eindhoven University of Technology, 5600 MB Eindhoven, The Netherlands; Institute for Complex Molecular Systems, Eindhoven University of Technology, 5600 MB Eindhoven, The Netherlands; Department of Biomedical Engineering, Eindhoven University of Technology, 5600 MB Eindhoven, The Netherlands; Institute for Complex Molecular Systems, Eindhoven University of Technology, 5600 MB Eindhoven, The Netherlands; Facultat de Medicina i Ciències de la Salut, Unitat de Biofísica i Bioenginyeria, Universitat de Barcelona, 08036 Barcelona, Spain; Department of Biomedical Engineering, Eindhoven University of Technology, 5600 MB Eindhoven, The Netherlands; Institute for Complex Molecular Systems, Eindhoven University of Technology, 5600 MB Eindhoven, The Netherlands; Department of Biomedical Engineering, Eindhoven University of Technology, 5600 MB Eindhoven, The Netherlands; Institute for Complex Molecular Systems, Eindhoven University of Technology, 5600 MB Eindhoven, The Netherlands; Department of Biomedical Engineering, Eindhoven University of Technology, 5600 MB Eindhoven, The Netherlands; Institute for Complex Molecular Systems, Eindhoven University of Technology, 5600 MB Eindhoven, The Netherlands

**Keywords:** morphometry, fibroblast, phenotype transition, myofibroblast, focal adhesions

## Abstract

The switching of the fibroblast phenotype to myofibroblast is a hallmark of a wide variety of tissue pathologies. This phenotypical switch is known to be influenced not only by humoral factors such as TGF-β, but also by mechanical and physical cues in the cellular environment, and is accompanied by distinctive changes in cell morphology. However, the causative link between these cues, the concomitant morphological changes, and the resulting phenotypic switch remain elusive. Here, we use protein micropatterning to spatially control dermal fibroblast adhesion without invoking exogenous mechanical changes and demonstrate that varying the spatial configuration of focal adhesions (FAs) is sufficient to direct fibroblast phenotype. We further developed an automated morphometry analysis pipeline, which revealed FA eccentricity as the primary determinant of cell-state positioning along the spectrum of fibroblast phenotype. Moreover, linear fibronectin patterns that constrain the FAs were found to promote a further phenotype transition, characterized by dispersed expression of alpha-smooth muscle actin, pointing to an interesting possibility of controlling fibroblast phenotype beyond the canonical fibroblast–myofibroblast axis. Together, our study reveals that the spatial configuration of adhesion to the cellular microenvironment is a key factor governing fibroblast morphotype and phenotype, shedding new light on fibroblast phenotype regulation.

**Significance Statement**

Within living tissues, the change in the characteristics of fibroblasts is considered to be a hallmark of the healthy or pathological state of the tissue. However, the mechanism by which the biophysical properties of the tissue regulate fibroblast characteristics is poorly understood. In this study, we combine quantitative analyses, including image morphometry, RNA profiling, and mechanical measurements, to demonstrate that fibroblast adhesion morphology plays a deterministic role in triggering fibroblast activation. Moreover, our work uncovers the existence of a previously unknown phenotype of dermal fibroblasts, referred to as matrifibrocytes. This direct link between fibroblast adhesion, morphology, mechanical properties, and phenotype may offer new strategies to control and modulate fibroblast behavior and, therefore, tissue physiology.

## Introduction

Fibroblasts are the key cellular players in the maintenance and restoration of homeostasis within tissues, for example, during wound healing in the skin dermis. They physically and chemically remodel the environment mainly by depositing and degrading extracellular matrix (ECM) components, such as fibronectin and collagen (1, 2). Following injury, an inflammatory response and disturbed tissue homeostasis result in the activation of fibroblasts (3, 4). A uncontrolled fibroblast activation, in turn, leads to an imbalance in ECM regulation, causing a partial or complete loss of function of the tissue, eventually resulting in a severe fibrotic response, as exemplified in pulmonary, cardiac, and hepatic fibrosis (5–9). In vivo, inflammation leads to the release of humoral/chemical factors, such as TGF-β_1_, which can trigger fibroblast activation. Indeed, the role of TGF-β_1_ in eliciting fibroblast activation is well documented and has been widely studied in vitro (10–12). More recently, it has also been shown that physical and mechanical cues in the microenvironment, such as stiffness, topography, and geometry, also have a strong influence on fibroblast activation (13, 14). This suggests a link between the mechanical regulation of fibroblast phenotype and their ability to sense and transduce mechanical cues in their environment. Despite the obvious biomedical relevance in a variety of tissue pathologies, the biophysical mechanism underlying such a potential link and its relation with the well-known humoral activation of fibroblast are poorly understood.

Under physiological conditions, fibroblasts possess a spindle-shaped morphology. Upon activation, the normally spindle-shaped fibroblasts rearrange their cytoskeletal organization with a denser network of actin stress fibers traversing the complete cell length. This allows the cells not only to acquire a more migratory behavior, but also to release ECM components, leading to a mechanically stiffer environment within the tissue (15). Furthermore, the enhanced formation of actomyosin stress fibers allows for the incorporation of α-smooth muscle actin (αSMA) within the actin fiber network, enhancing the cell's contractility (3, 15). This morphological alteration and acquisition of a strongly contractile state are signatures of a myofibroblastic phenotype, which is canonically thought to be the end stage of fibroblast differentiation (16–18). Myofibroblasts exhibit more pronounced proliferation and ECM deposition, which are essential to promote tissue repair. However, this increased ECM deposition also carries the risk of excessive structural and mechanical changes, for example, leading to fibrosis (7, 8, 15). Ensuring balance of phenotypical states within this fibroblast–myofibroblast spectrum is therefore critical for restoring tissue homeostasis.

The activation of fibroblasts involves changes in cytoskeletal composition and organization, cell morphology, and contractility, which simultaneously play an important role in the cell's ability to sense mechanical cues in their environment (15, 19, 20). This connection is especially exciting as it may suggest a therapeutic strategy aimed at controlling fibroblast phenotype by modulating the ECM properties. Interestingly, recent evidence suggests that variations in collagen network properties (i.e. pore size and fiber thickness) affect myofibroblastic differentiation of adipose stromal cells (20). Additionally, it has been proposed that collagen organization is a key factor to induce fibroblast differentiation (17). For adherent cells, such as fibroblasts, mechanosensing starts with the binding of cell membrane receptors, particularly integrins, to ligands in the ECM, forming focal adhesions (FAs). The establishment of FAs subsequently leads to cytoskeletal rearrangements, allowing for intracellular transduction of mechanical and physical stimuli (21–23). Consistent with the idea that mechanical interactions with the substrate and cell phenotype are interlinked, inhibition of Rho kinase, which is important for the maturation of FAs, was shown to cause a minor fibroblasts phenotype shift (24). However, to date, the causal link between FA-dependent cell–substrate interactions and the phenotypical state of fibroblasts has not been explored.

In this study, we address this question by systematically decoupling the humoral, spatial, and mechanical stimulation routes of fibroblast phenotype regulation. Firstly, we demonstrate that by manipulating the spatial distribution of FAs, by means of light-based protein micropatterning, human dermal fibroblast phenotype can be controlled, even in the absence of humoral stimulation. Secondly, we developed an automated image analysis pipeline, which enables the robust characterization of dermal human fibroblast morphological state (“morphotype”) along the spectrum of fibroblast phenotypes. Finally, we correlated these to the mechanical properties (“mechanotype”) and gene expression profiles (“genotype”) of the human dermal fibroblasts. Combined, these analyses identified an important link between the spatial regulation of cell–substrate adhesion complexes and downstream cell morphotype, genotype, mechanotype, and phenotype. Taken together, this offers a new perspective for potentially modulating tissue physiology by manipulating microscale properties at the cellular level.

## Results

### Fibroblast morphology is affected by humoral and contact cues

**Fig. 1.**
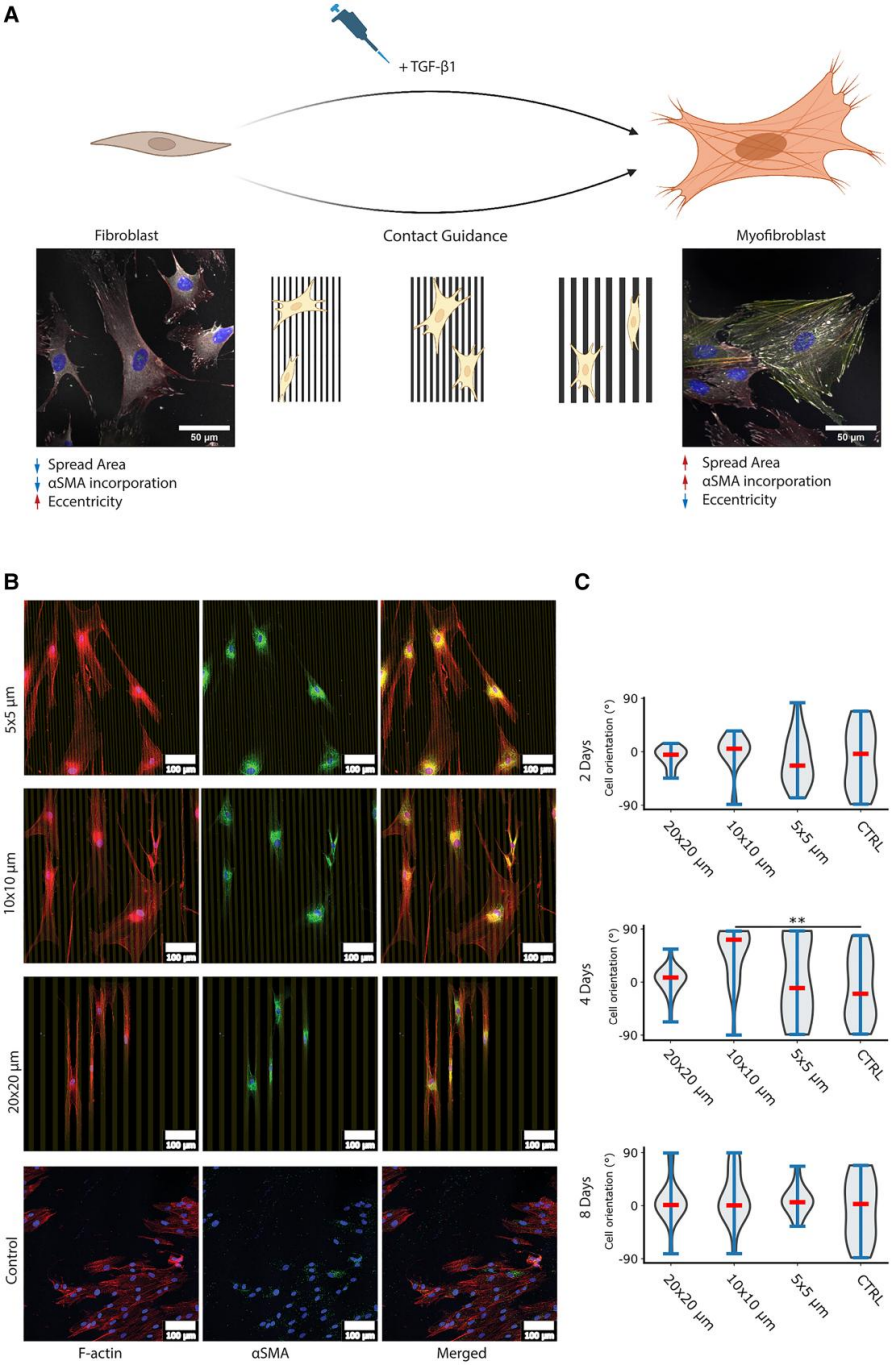
Modulation of fibroblast morphology by humoral and contact cues. A) Activation of fibroblasts into myofibroblasts is known to be induced by humoral/chemical stimulation, for example, by TGF-β. This phenotype transition results in certain morphotypical changes, such as an increase in cell spread area, an increase αSMA incorporation into the stress fibers, and a reduction in cell elongation. Here, we ask whether this phenotype transition can also be induced by modulating cell morphotype through contact guidance. Cells were stained for DAPI, f-actin, vinculin, and αSMA. Scale bar: 100 μm. B) Fluorescence images of human dermal fibroblast cultured for 8 days on linear fibronectin patterns (shown in gray) of different dimensions (width × spacing). C) The orientation distribution of cells on different patterns and at different time points, shown as violin plots where 0° indicates parallel alignment to the protein micropattern, while 90° and −90° indicate perpendicular orientation of the cells with respect to the protein micropattern. Data are taken from >15 cells from 12 independent samples per condition, **P < 0.05.

Conventionally, fibroblast-to-myofibroblast transition (FMT) is known to be triggered by humoral factors, particularly TGF-β, which is released during inflammatory response. As a cell model to investigate FMT, here we used human dermal fibroblasts, whose phenotypic activation is crucial in wound healing and skin tissue regeneration (18, 25) and is known to be regulated by humoral and mechanical factors (7, 24). The addition of 10 ng/mL TGF-β to an in vitro culture of human dermal fibroblasts under serum-starved condition for 4 days indeed led to clear myofibroblast signatures, such as the incorporation of αSMA within the cytoskeleton stress fibers (Fig. 1A), consistent with previous reports (24, 26–28). The effect was dependent on the concentration of TGF-β (SI Appendix, Fig. S1), demonstrating that fibroblast phenotype during FMT lies along a spectrum rather than in binary states. Furthermore, the incorporation of αSMA within the stress fibers was accompanied by concomitant changes in the cell shape and cytoskeletal organization, although the causal relation between the two, if any, is unknown. In this study, we hypothesized that FMT can alternatively be induced through modulation of the cell's morphological state (“morphotype”) and extent of elongation. To test this, we generated substrates with linear cell adhesive patterns (Fig. 1A) using light-induced molecular adsorption of protein (LIMAP) method (29, 30). This approach allowed us to directly probe the relevance of cell elongation and spatial distribution of cell–substrate adhesion, and decouple it from other humoral and mechanical cues. The linear fibronectin patterns with equal widths and spacings of 20, 10, and 5 µm were stable over the duration of the experiment (SI Appendix, Fig. S2). Hereinafter, for brevity, we will use the notation of width × spacing to refer to the different fibronectin linear patterns (i.e. “20 × 20 lines” refers to a fibronectin pattern with 20 µm width and 20 µm spacing). These pattern dimensions were chosen as earlier studies from our group showed these dimensions to evoke strong contact guidance and cell reshaping (29, 31, 32).

The protein patterns were found to influence the overall cell morphology and orientation (Fig. 1B). When grown on homogeneously fibronectin-coated surfaces, human dermal fibroblasts kept their spindle-shaped form; the presence of αSMA was minimal and there was no observable integration into the actin cytoskeleton, as expected. After 8 days of culture on the linear fibronectin patterns, the cells showed a higher spread area with a more pronounced expression of αSMA, which was incorporated within the actin stress fibers. It is interesting to note that on the 20 × 20 lines, the fibroblasts were more elongated compared with the smaller lines, where the cells exhibited a more spread-out morphology (Fig. 1B). Consistent with an earlier study (32), this is likely attributable to the balance between the cell's energetic tendency to maximize spread area while avoiding gaps in between the linear fibronectin-coated areas. Additionally, it resulted in anisotropic distribution of cell orientation toward the direction of the linear protein micropatterns. To check this and to assess at what time points the micropatterns influenced cell organization, we quantified individual cell orientation at days 2, 4, and 8 (Fig. 1C). We observed that 2 days of culture on the protein micropatterns were enough to induce preferred orientation of the cells (*P* = 0.0030) and 20 × 20 lines evoked the strongest orientation response compared with 10 × 10 and 5 × 5 lines. Moreover, the induced orientation response became weaker at later time points (4 and 8 days). After 8 days, only 20 × 20 lines still resulted in orientation anisotropy compared with on homogeneous control (*P* = 0.0155). Overall, we found that, using protein micropatterns, we successfully manipulated the cell–substrate adhesion, fibroblast morphology and orientation, and intracellular organization.

### Morphometric analysis reveals FAs to be a key determinant of contact-dependent fibroblast morphotype

**Fig. 2.**
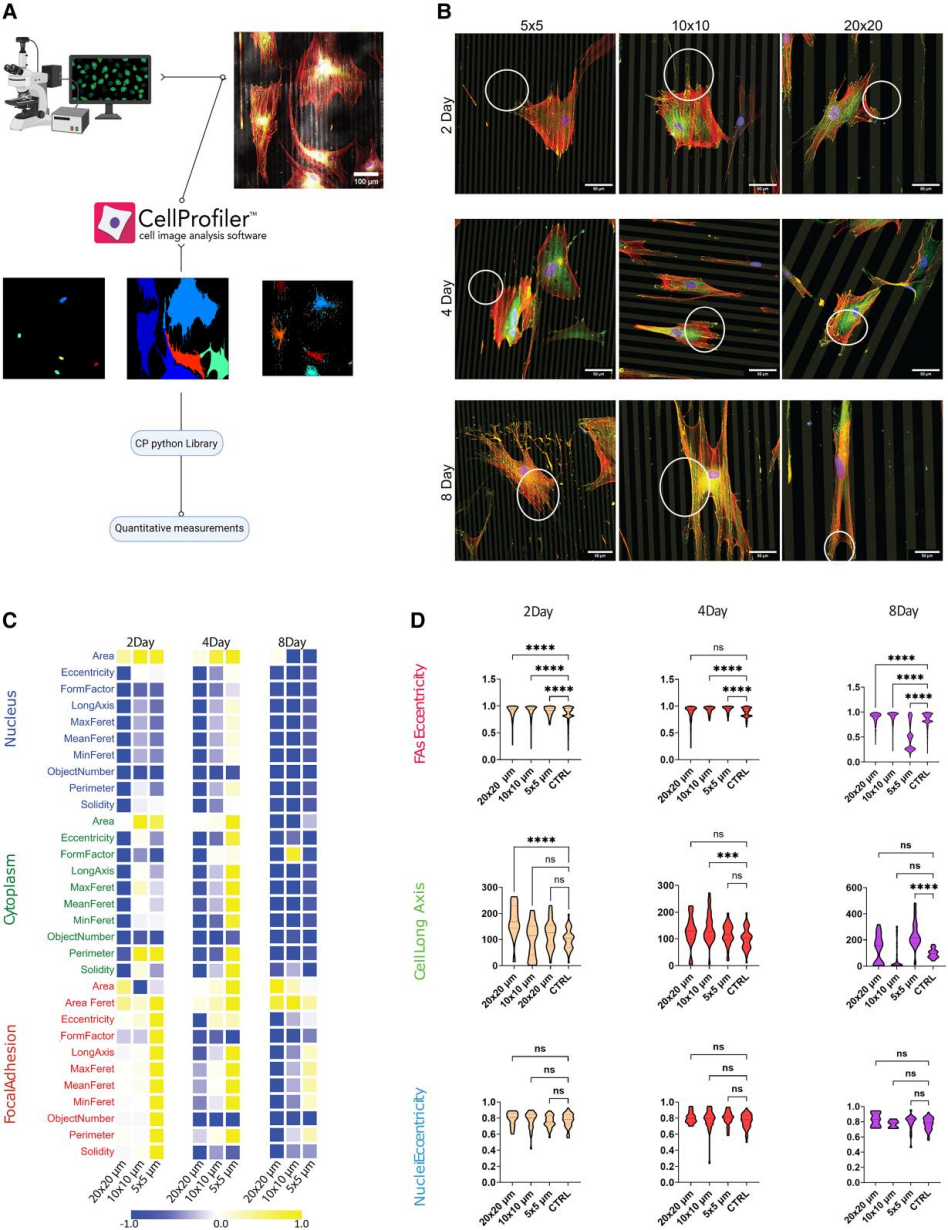
Morphometric characterization of fibroblasts on contact cues. A) An overall schematic of the automated image analysis workflow from image acquisition (scale bar: 50 μm) to the extraction of quantitative morphometric measurements. See SI Appendix, Fig. S3 for a detailed description. B) Representative examples of single cells stained for nuclei, F-actin, and vinculin on the different protein micropatterns (gray) at different time points. White circles indicate prominent FAs around the nuclei and at the cell periphery; control conditions are shown in Fig. S4. Scale bar: 50 μm. C) A heat map of normalized morphometric parameters. Data were normalized against the mean value of the control condition (i.e. fibroblasts seeded on homogeneous fibronectin-coated coverslips). D) A comparison of the distributions of the three most critical morphometric parameters (selected from the SHAP analysis; see SI Appendix, Fig. S5) between different protein micropatterns. Data are taken from 10 to 20 cells and 10 independent samples per condition. ***P < 0.005, ****P < 0.0005 (one-way ANOVA).

One of the most known signatures of FMT is a change in the overall cell morphology, such as cell area and perimeter. However, this change has been largely qualitatively described and never quantitatively compared. It is, therefore, important to establish key morphotypical signatures of fibroblast phenotypes, especially as we sought to understand how contact cues affect the cell's phenotypical state. Another challenge is heterogeneity within the cell population, either of biological or of entropic origins (33–35), which implies that a large number of (high-resolution) cell morphologies need to be analyzed to extract any statistically meaningful parameter. To address these challenges, we developed an automated image analysis pipeline that makes use of preexisting open-source tools (i.e. CellProfiler and Python libraries) to efficiently carry out the morphometric analysis of a large number of high-resolution microscopy images (Fig. 2A; see also the more detailed analysis workflow in SI Appendix, Fig. S3). Using this tool, we analyzed >500 individual, high-resolution cell morphologies on different contact patterns and time points (SI Appendix, Fig. S4).

Qualitatively, we observed that, after 2 days on the protein micropatterns, fibroblasts on the 20 × 20 lines achieved a higher degree of elongation compared with those on the smaller micropatterns (Fig. 2B, top panel). This cell body elongation was accompanied by an increase in the quantity and size of FAs, which were located not only at the cell periphery but also near the nuclei (Fig. 2B, circles). After 4 days, the differences between the different micropatterns became more prominent. This was particularly true on the 10 × 10 lines, where the cells exhibited a higher degree of elongation, a more prominent formation of stress fibers, and more FAs compared with on day 2 (Fig. 2B, middle panel). After 8 days, the cells on the 5 × 5 and 10 × 10 lines were observed to fill the gaps between the cues, allowing for more cellular spreading. On the other hand, cells on the 20 × 20 lines showed strong elongation but with fewer and smaller FAs. Interestingly, cells on 20 × 20 lines showed a different morphology compared with the control, with a higher degree of cell alignment to the pattern despite the absence of αSMA (Fig. 2B, bottom panel). Based on these qualitative observations, we identified a total of 40 main morphometric parameters, at the levels of whole cells, nucleus, and FAs, distinguishing the fibroblast morphology on various protein micropatterns. We went on to quantify these morphometric parameters using our image analysis pipeline (SI Appendix, Fig. S3, and Table S2).

The morphometric image analysis was fed with fluorescence images of the whole cells (cytoskeleton staining), nuclei (DAPI staining), and FAs (vinculin staining). A summary of selected morphometric parameters is shown in Fig. 2C in the form of a heat map, as normalized with respect to the corresponding parameter values for the control conditions (i.e. 0 for fibroblasts on homogeneous fibronectin coating and 1 for fully activated myofibroblasts). In line with our earlier qualitative observations, we found that after 2 days on the protein micropatterns, most morphometric parameters of the detected objects (i.e. nuclei, cytoplasm, and FAs) were enhanced compared with those in the control fibroblasts. After 4 days, this difference became even more pronounced, in particular for the FA parameters. The FA eccentricity, for example, on 10 × 10 lines, was significantly higher (0.92 ± 0.06, *P* < 0.0005) with respect to the control (0.88 ± 0.08), indicating strong cell adhesion. Remarkably, on day 8, there was a clear switch in the morphometric parameters of the nuclei and cell body. These parameters were drastically decreased, rather than enhanced, with respect to the control fibroblasts. Cell perimeter decreased on the protein patterns, where it became three times smaller than in the control fibroblasts. At the level of the FAs, the morphometric properties for the 10 × 10 and 5 × 5 lines were higher with respect to the control fibroblasts but were significantly decreased on 20 × 20 lines, both with respect to the previous time points and with respect to the control fibroblasts. The average FA perimeter was lower, 1.16 μm, than the fibroblasts cultured on homogeneous coating, 2.36 μm. On the other hand, after 8 days of culture, we observed a similar behavior between the control fibroblasts and on the 20 × 20 lines, where the cells presented a smaller but very elongated cell body, causing a strong nuclei elongation as well. Moreover, the cell dimensions decrease with prolonged culture time. In the latter case, cells presented a decreased number of FAs within the cell body, which was also significantly different from the expected fibroblast phenotype.

We next asked which of the independent quantitative parameters were most strongly responsible for describing the different morphotypes of fibroblasts on different contact patterns. To answer this, we run a SHapley Additive exPlanations (SHAP) analysis (SI Appendix, Fig. S5), which is a commonly used tool quantifying feature contributions in predictive models in machine learning (36). Interestingly, for all experimental conditions, three parameters were consistently identified in the SHAP analysis to be the strongest contributors: FA eccentricity, cell long axis, and nuclei eccentricity (SI Appendix, Fig. S5). The strongest contributor is FA eccentricity, quantifying the extent of elongation of the FAs, which was found to be higher on the protein micropatterns than on control substrates after 4 days of culture (Fig. 2D, *P* = 0.0049). It is notable that the three strongest contributors associated with the degree of object elongation, at the level of the FAs, nuclei, and cell bodies. Together, this quantitative analysis of fibroblast morphology shows that contact cues in the form of protein micropatterns are sufficient to control fibroblast morphotype through the regulation of FAs.

### Contact cues trigger the fibroblast phenotype shift

**Fig. 3.**
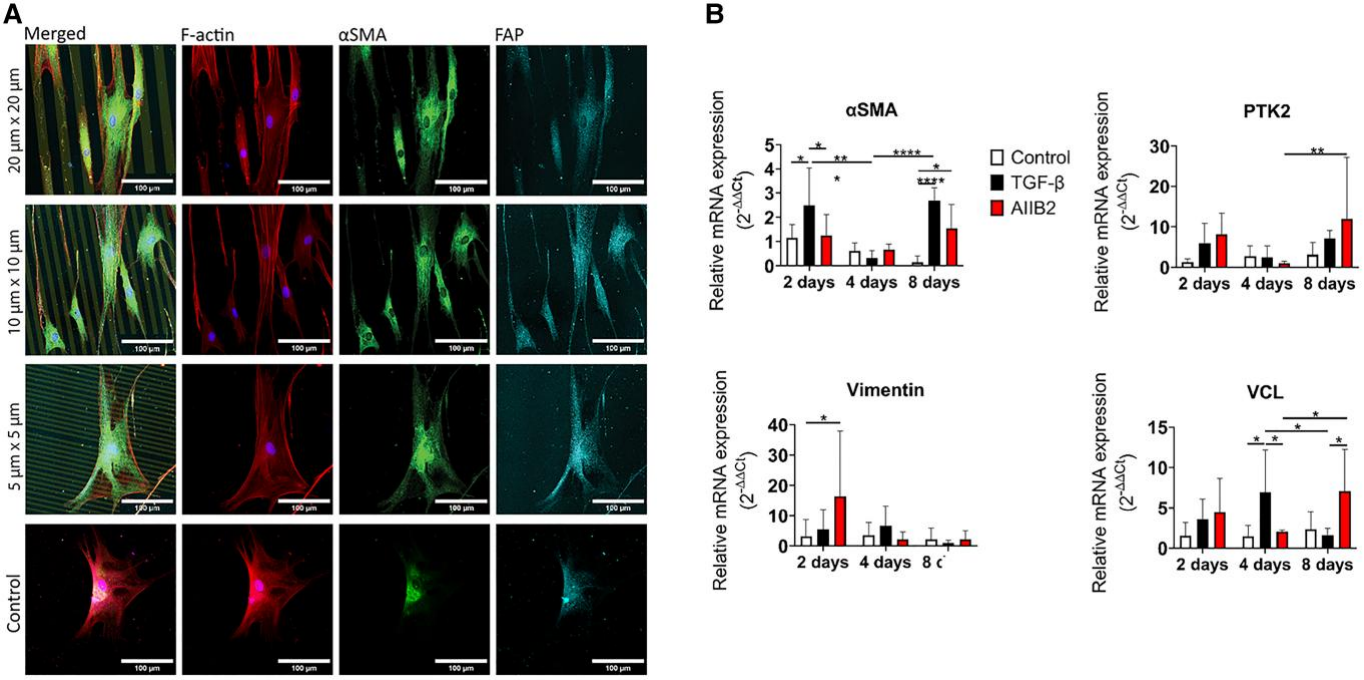
Characterization of fibroblast phenotype shift. A) Fluorescence images of fibroblasts cultured for 8 days on different patterns. The αSMA is only present in the cytoplasm and not incorporated within the cytoskeleton, although the cell activation can be appreciated from the FAP staining. B) qPCR data for αSMA, vimentin, vinculin (VCL) and FA kinase (PTK2). *P < 0.5, **P < 0.05, ****P < 0.0005.

Next, we asked whether the observed fibroblast morphotypes are linked to specific phenotypes along the fibroblast–myofibroblast axis. To study this, we examined the known phenotypical signatures of FMT at the protein and transcriptional levels. First, we investigated the degree of alignment of F-actin and αSMA. Colocalization of F-actin stress fibers (which are only present when the fibroblasts are in an activated state) and the αSMA fibers (which are only present in myofibroblasts) has been generally used to discriminate the two phenotypes (5, 17, 18, 35). Additionally, we analyzed the images using FOAtool, an image-based algorithm developed specifically to quantify and analyze filamentous structures (37). The microscopy images highlighted a decrease of F-actin cytoskeleton stress fibers in fibroblasts on a protein micropattern compared with fibroblasts cultured on a homogeneous substrate (Fig. 3A), consistent with the previous report (38). Moreover, the cytoskeleton showed higher alignment to the protein patterns on 20 × 20 lines compared with on the 10 × 10 and 5 × 5 lines (SI Appendix, Fig. S6). Since αSMA seemed to be present but not incorporated within the stress fibers on day 8, we further examined fibroblast activation protein (FAP), which has been widely used for screening for activated fibroblasts and cancer-associated fibroblasts (39). We observed FAP colocalization with αSMA (Fig. 3A), underlining the activated stage of the fibroblasts after 8 days of culture on the protein micropatterns.

To validate this shift in phenotype, we tested and compared the transcriptional expression of αSMA, together with the main markers used to quantify FAs formation, the structural protein vinculin (VCL) and the FA kinase (PTK2; Fig. 3B and SI Appendix, Fig. S7). Treatment with TGF-β to achieve myofibroblast differentiation resulted in the expected initial increase in expression of αSMA, which then lowered at day 4. This reduction in αSMA at day 4 coincided with a marked increase in expressions of VCL, PTK2, and vimentin, which were comparable with the expression levels observed after 2 days of activation. Interestingly, after 8 days of TGF-β treatment, the transcriptional expression pattern observed in the cells was similar to the activated but not fully differentiated state on day 2. Clear differences could also be appreciated in the markers involved in FA formation (e.g. PKT2, VCL). To further confirm the shift in fibroblast phenotype, we analyzed vimentin, whose expression is known to be increased upon myofibroblastic differentiation (39). As expected, TGF-β was found to increase the expression of vimentin on day 2; however, to our surprise, the expression of vimentin decreased over time.

Finally, we tested the role of cell–substrate contact cues on this phenotypic shift by adding 10 ng/mL AIIB2, an inhibitor of β1 integrins binding. The inhibitor successfully prevented the maturation of FAs and prevented proper cell motility. The formation of FAs and the network of stress fibers are effectively prevented by AIIB2, which ultimately obstructed cells from undergoing FMT and incorporating αSMA (SI Appendix, Figs. S8 and S9). In the presence of AIIB2, the mRNAi expression levels of αSMA in the early time points were similar to those in the control condition. However, unlike the control condition, the expression levels increased over time. The expression profiles of other proteins, such as PKT2 and VCL, exhibited similar trend after 2 days and reached even higher levels compared with the control condition and the activated fibroblasts after 8 days of culturing. As for vimentin, its mRNA level was higher than the control condition after 2 days of culturing but decreased in the next time points.

As a whole, these data indicate a phenotypic change of the fibroblasts that was induced not only by treatment with TGF-β, but also as a consequence of perturbation of integrin-mediated substrate adhesion with time. Furthermore, the analysis revealed mRNA expression profiles at prolonged culture that are atypical for either fibroblasts or myofibroblasts, suggesting a further phenotypic state beyond the canonical fibroblast–myofibroblast axis.

### Fibroblast phenotype shift results in changes in cell mechanical properties

**Fig. 4.**
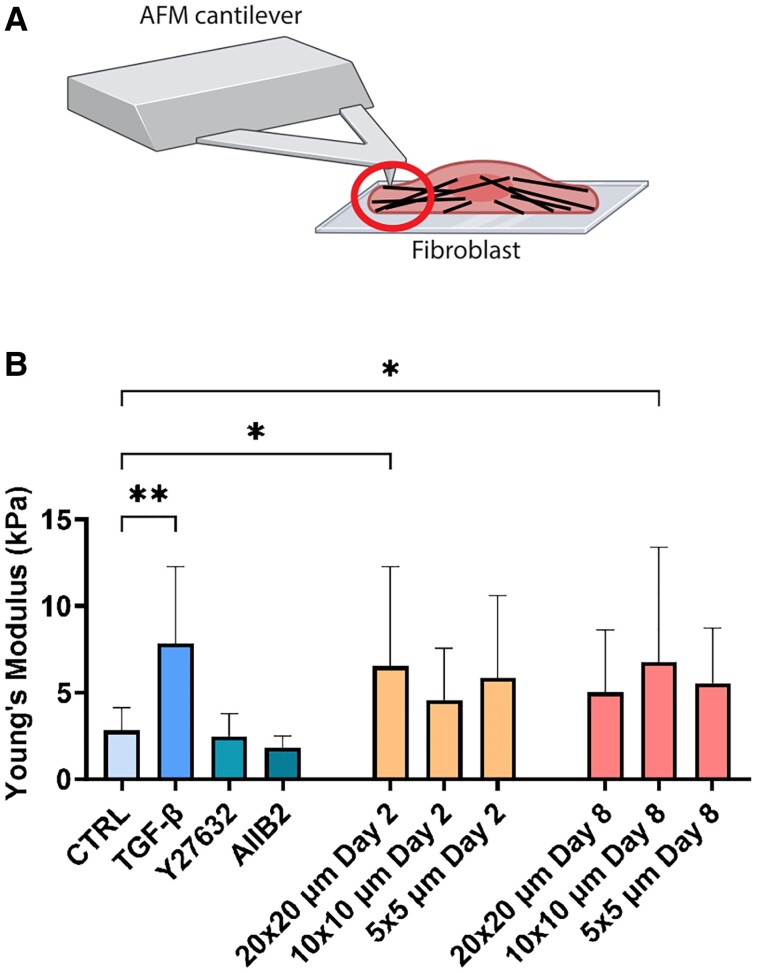
Mechanical characterization of different fibroblast phenotypes. A) Schematic of measurement of cell stiffness away from the nucleus (red circle) using AFM. B) Measured Young's modulus of fibroblasts at different conditions on day 2 (control, in the presence of TGF-β, Y27632, or AIIB2) and at different time points on fibronectin patterns of different sizes. Data are shown as mean ± SD from *n* ≥ 18 cells per condition. *P < 0.5, **P < 0.05.

The phenotypic changes due to the contact events that we observed, including in the cytoskeletal organization, stress fibers, and cell morphology, are known to affect the mechanical properties of the cell (40). Additionally, the cytoskeleton translates the mechanical and physical signals from the microenvironment into intracellular signals through mechanotransduction. In the context of FMT, this culminates in the incorporation of αSMA within the stress fibers, increasing cell contractility (8, 24, 41, 42). As such, we hypothesized that the phenotypic changes would manifest in changes in the cell mechanical properties. To test this hypothesis, we measured cellular stiffness using atomic force microscopy (AFM). In particular, we focused on measurements of the cytoskeletal regions (i.e. away from the nucleus), to extract information about the impact of the contact-induced phenotypic changes on the cytoskeletal mechanical properties (Fig. 4A and SI Appendix, Fig. S10).

First, we checked whether FMT induced by conventional humoral cues resulted in a change in cell stiffness by adding of TGF-β for 2 days. Indeed, we observed a ∼2.5-fold increase in the measured Young's modulus (Fig. 4B, *P* = 0.04). It has been shown that fibroblast phenotype shift caused by humoral changes can lead to quantifiable mechanical changes in the cell (43). To further test whether direct perturbation of the cell's mechanical machinery also resulted in such mechanical changes, we added ROCK inhibitor (Y27632) and integrin inhibitor (AIIB2) and observed that cell stiffness was slightly but not significantly reduced. We then tested whether phenotypic changes caused by contact cues also resulted in changes in cell mechanics. Indeed, the cells on the protein patterns were stiffer compared with the control condition and similar to the condition activated by TGF-β (Fig. 4B). Interestingly, after 8 days of culture, we observed significantly higher Young's modulus only on 10 × 10 µm lines (*P* = 0.02) and not on the other pattern dimensions. This is consistent with the different cell morphology on the protein patterns (Fig. 2). On 10 × 10 lines, the cells present defined actin cytoskeleton, while on 5 × 5 and 20 × 20, the network is almost absent. These results suggest that spatial contact cues trigger fibroblast activation by causing cytoskeletal rearrangements and changes in cell mechanics.

### Fibroblast phenotype shift is directly affected by the spatial positioning of FA

**Fig. 5.**
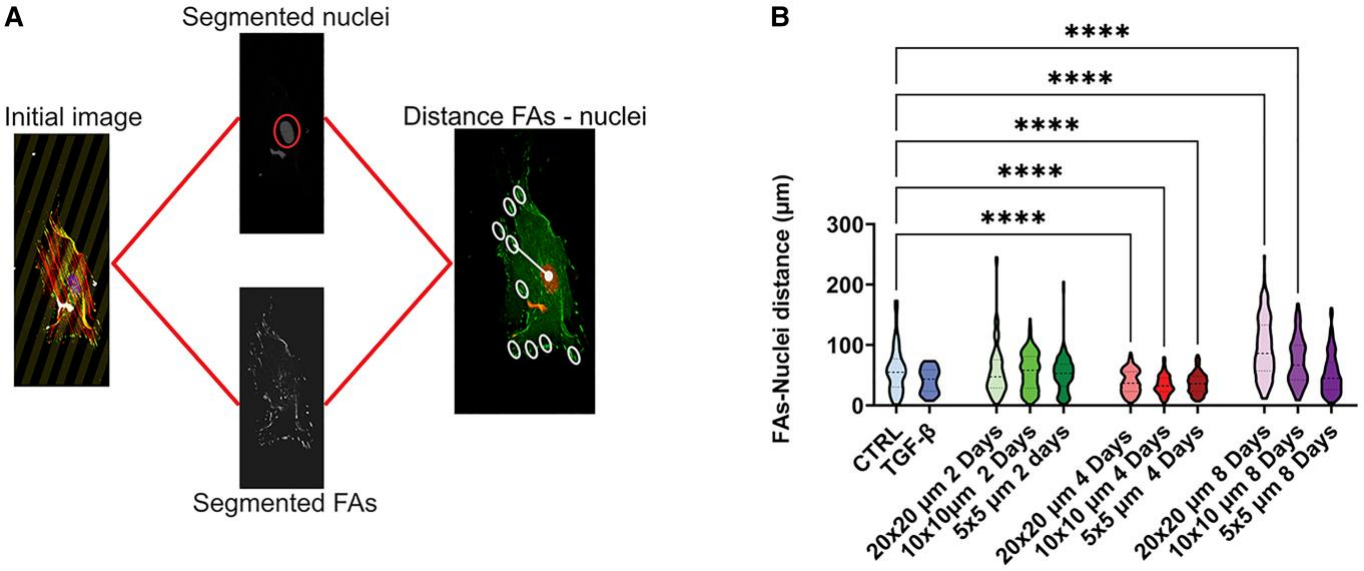
Spatial distribution of FAs during fibroblast phenotype shift. A) Scheme illustrating the image processing to measure the distance (white line) between the centroids of the detected FAs (empty white circle) and the nucleus centroid (filled white circle). B) Distribution of measured distance between FAs and nucleus centroids on different protein micropatterns on days 2, 4, and 8 (control conditions in the presence and absence of TGF-β). ****P < 0.0005.

So far, our findings have pointed to the impact of contact cues on fibroblast phenotype, morphotype, and mechanotype. While our earlier analysis demonstrated the importance of the *morphological features*, such as FA eccentricity, we further asked whether such impact is also implicated through nonmorphological *spatial positioning* of the FAs, which mediate not only the cell morphology but also mechanical properties. In other words, is contact-induced fibroblast phenotype shift regulated through morphological changes or mediated by the spatial configurations of the FAs? To answer this question, we analyzed the distance between the centroids of the segmented FAs and the segmented nuclei of the same cells (Fig. 5A). We found that, after 2 days, fibroblasts present on average larger (51 μm) distance between FAs and nuclei with respect to the TGF-β-activated myofibroblasts (41 μm, *P* = 0.0001), despite the fibroblasts' smaller cell area. On day 2, fibroblasts cultured on protein patterns showed similar distances between FAs and nuclei compared with the fibroblasts cultured on a homogeneous substrate; on the other hand, the FAs were dispersed throughout the cell body, resulting in a low mean distance (34–39 μm, *P* = 0.0001) between FAs and nuclei at day 4 (Fig. 5B). Interestingly, on day 8, the elongated cells exhibited a higher average distance (95 μm on the 20 × 20 lines, *P* = 0.0001) between the FAs and the nuclei (Fig. 5B). These results show that the distance of FAs from the nuclei changes with respect to the cells activation and phenotypical change.

## Discussion

Fibroblasts generally maintain their phenotype until the steady state of the tissue is disrupted, for example, in the case of the inflammatory response during wound healing. At this stage, several humoral factors, such as TGF-β, are released that cause the activation of the fibroblasts (13, 24, 44). This fibroblast activation results in changes in phenotypic behavior, such as higher motility, allowing the activated fibroblasts (often referred to as proto-myofibroblasts) to reach regions of interest by migrating on a fibronectin bed in the tissue using integrin-based adhesion mechanism (13, 28). The proto-myofibroblasts then complete the phenotype transition into myofibroblasts through the incorporation of αSMA within the cytoskeleton, enhancing their contractile capability, and the deposition of different ECM components, leading to a change in the tissue mechanical properties (14, 43, 44). In this work, we hypothesized that this complex sequence of events is spatially and morphologically mediated by contact cues between the cells and the extracellular environment, even in the absence of stimulatory humoral factors. In other words, we suggest a direct coupling between fibroblast phenotype, morphotype, and mechanotype as mediated by spatial contact cues.

To test this hypothesis, it is important to use an analytical tool that allows robust quantification of cellular morphometrical parameters. Several tools are available (45, 46); however, most of them require manual tuning of the image processing parameters by the operator, limiting the degree of objectivity. In addition, many run on proprietary software, are not adjustable (i.e. functioning as a black box), demand extensive computational resource, or are not suitable for batch processing of large image files across different experimental conditions. To tackle these limitations, we developed an automated image processing pipeline, making use of two open-source and widely used software: Python and CellProfiler, using custom-written Python packages and CellProfiler pipelines. This approach perfectly combines the versatility of Python for deep processing and analyzing and the suitability of CellProfiler for morphometric analysis of (batches of) cell biological images. The batch of images are automatically and consistently preprocessed in Python and analyzed for the morphometric parameters in CellProfiler, with the possibility for operator examination and intervention in critical steps. The output batch of data is handled and further postprocessed automatically (e.g. for statistical analyses) using Python to optimize user-friendly data findability, accessibility, interoperability, and reusability (i.e. FAIR data principle (47)). Furthermore, the workflow also includes built-in visualization and analysis module, such as the SHAP analysis, which can assist the operator in identifying the critical parameters of interest. To demonstrate the utility of this tool, here we analyze 40 morphometric parameters of fibroblasts at the whole-cell, nucleus, and FA levels as they undergo phenotypic shift induced by contact events.

Using this tool, we aimed to correlate the morphotype and phenotype of fibroblast along the FMT. When not in an activated state, fibroblasts are spindle-shaped, presenting a low amount of actin stress fibers together with low or absent expression of αSMA. They are generally smaller with a weaker formation of FAs (48). FAs that do form are mostly found at the cell periphery, at the farthest spots from the nucleus. These suggest a correlation between FA-based mechanosensing and fibroblast phenotype. Indeed, Sapudom et al. (48) recently achieved fibroblast activation by controlling the anisotropy of collagen fibrils on which the cells are cultured. In our study, we used protein micropatterns that allow more precise control of cell–substrate adhesion and similarly found that substrate anisotropy influences fibroblast phenotype. Importantly, we found that the phenotypic regulation is mediated by changes in cellular morphotype and mechanotype. Out of the morphometric parameters that we screened, FA eccentricity was identified as the key determining parameter. In addition, quantification of the localization of FAs within the cells revealed that FAs in fibroblasts are located further away from the nucleus than in activated myofibroblasts, further suggesting the involvement of force generation and cell contractility, and therefore also the mechanical properties of the cells. Indeed, mechanical measurement of the cells indicated that contact guidance induces off-nuclei fibroblast stiffening comparable with the fibroblast activation triggered by the presence of TGF-β. These findings suggest that substrate adherence cues in the form of protein micropatterns may lead to a change of the mechanical state or stress in the cells, which in turn triggers fibroblast activation. Specifically, the micropatterns cause the cells to elongate and rearrange the mechanosensing machinery, leading to different cytoskeletal structures and redistributing the mechanical stresses through the contact points with the substrate. We further asked whether this cell–substrate adhesion is important for fibroblast regulation not only at the morphological and functional levels but also at the genetic level. Our qPCR data clearly showed differences in mRNA quantity at different phenotypical stages. During the myofibroblast stage, the mRNA present was lower while the stabilizing FA protein VCL increased in comparison with the control condition. However, at a longer culturing time point, when the cells appear to be losing the myofibroblastic phenotype, the mRNA of αSMA increased drastically while the one from VCL showed a significant drop. Taken together, these data confirm that spatial contact cues at the cell–substrate interface can regulate fibroblast phenotype and genotype through modulation of cell morphotype and mechanotype. While in this study, we have focused on linear substrate patterns to interrogate the impact of elongated cell morphologies, it will be interesting in the future to also probe other types of protein patterns that can induce different extent of FA eccentricities and FA spatial distributions.

Our previous discussions depart from the assumption of a conventional fibroblast–myofibroblast phenotypic axis in FMT (5–8). However, our phenotype, genotype, and morphotype analyses interestingly also identified a phenotypic state of dermal fibroblasts at day 8 of our culture conditions that is distinct from the fibroblastic and myofibroblastic states, characterized by the presence of αSMA without incorporation into the cytoskeleton (Fig. S11), together with smaller and less numerous FAs. Intriguingly, these observed signatures of dermal fibroblasts are reminiscent of a particular and rarely reported phenotypic state of cardiac fibroblasts called matrifibrocytes (38). Cardiac matrifibrocytes have been reported to develop from long differentiation (8–10 days) of cardiac myofibroblasts, where the cells lose their contractility capability due to the disappearance of αSMA and FAs. Hitherto, matrifibrocytes have, to our knowledge, only been reported in the context of cardiac fibroblasts. Our observation of matrifibrocyte-like phenotype in dermal fibroblasts may suggest their more general relevance in maintaining tissue homeostasis in other tissues as well, for example, in the formation and regulation of scars during wound healing, as proposed earlier (38). Moreover, the possibility of controlling fibroblast phenotype and their role in physiology using spatial cues even beyond the canonical FMT axis is exciting and should be further investigated.

**Fig. 6.**
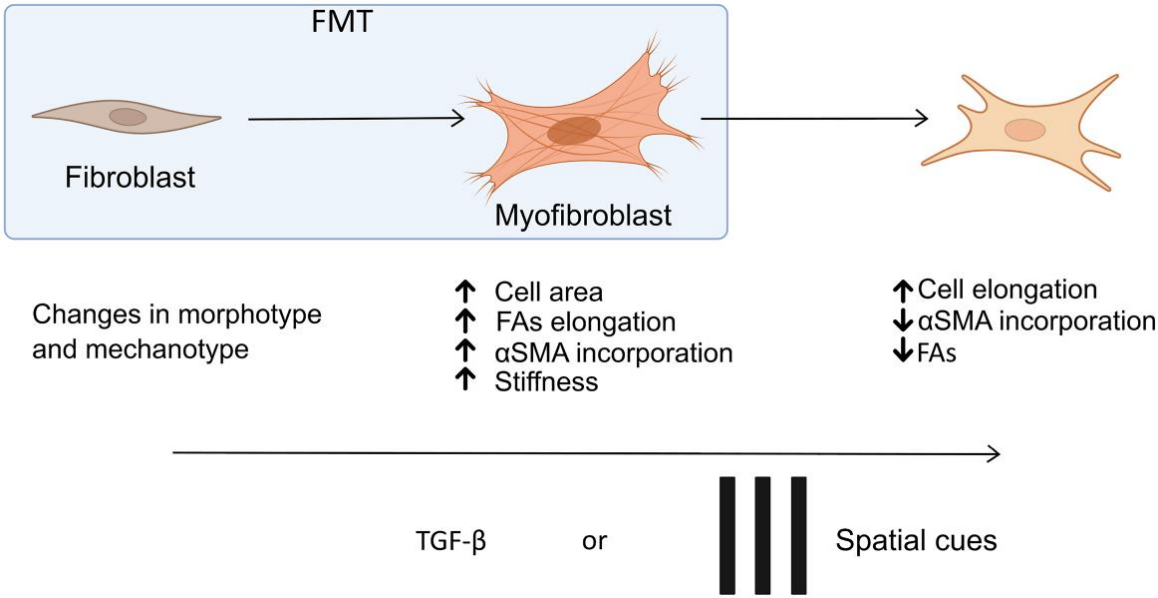
Spatial contact-dependent regulation of fibroblast phenotype. The diagram illustrates how fibroblasts can be activated by contact-based protein micropatterns, and how the addition of TGF-β growth factor can induce further phenotypical stages beyond the myofibroblast (SI Appendix, Fig. S11).

This study aimed to investigate the mechanobiological regulation of fibroblast phenotype, which is a crucial process in tissue homeostasis. The use of protein micropatterning allows spatial control of dermal fibroblast adhesion and demonstrates that varying the spatial configuration of FAs is sufficient to trigger fibroblasts activation, irrespective of the presence of humoral stimuli, such as TGF-β. Our findings reveal that the spatial configuration of FAs with the cellular microenvironment, together with cell mechanics, is critical in governing fibroblast morphotype and phenotype, shedding important light on fibroblast regulation in tissue homeostasis (Fig. 6). Additionally, we showed the link between cell response to spatial cues and phenotype and revealed FA eccentricity as a key morphometric determinant of cell-state positioning along the spectrum of fibroblast phenotypes. Finally, the study highlights the importance of automated image analysis techniques in quantifying morphotypes of cells and demonstrates the potential of controlling fibroblast phenotype beyond the canonical fibroblast–myofibroblast axis. Overall, the study provides important insights into fibroblast regulation and may have significant biomedical relevance in tissue homeostasis and a variety of tissue pathologies.

## Materials and methods

### Cell culture

Normal Human Dermal Fibroblasts (nhDFs, Lonza, CC-2511) were expanded in Dulbecco's Medium Eagle's Medium (DMEM 41966-029, Thermo Fisher) with 10% fetal bovine serum (FBS; Biochrom AG) and 1% penicillin/streptomycin (Biochrom AG). The culture media was changed from the culture flask 1 day before the beginning of the experiment with DMEM containing 3% FBS and 1% P/S. Cells were seeded with a cell density of 471 cells/cm^2^ on 13 mm diameter patterned substrate glass coverslips in DMEM with 3% FBS and 1% P/S. Upon resuspension, 200 µL of cell suspension was pipetted on top of the glass surface to constrain the adhering cell regions and incubated at 37 °C for 30 min. As soon as cell alignment to the pattern was noticed, the well was filled with medium to remove nonadherent cells. The nhDF cultures were incubated for the duration of the different experimental conditions at 37 °C in a cell culture incubator, with 5% CO_2_.

### Protein micropatterning

The protein-micropatterned substrates were produced following the protocol of the maskless LIMAP method (29, 30). Coverslips were first activated using O_2_-plasma (30 s at 20 W) and incubated for 30 min with PLL (poly-L-Lysine, P4707; Sigma–Aldrich) at room temperature. The substrates were then washed three times with 0.1 M HEPES (4-(2-hydroxyethyl)-1-piperazineethanesulfonic acid) (8.0 < pH < 8.5), incubated at room temperature with mPEG-succinimidyl valerate (mPEG-SVA, 50 µg/mL in 0.1 M HEPES [8.0 < pH < 8.5], MW 5,000 Da, Laysan Bio) for 1 h, and washed again using phosphate-buffered saline (PBS) three times. After the samples dried, a photocleavage agent (4-benzoyl benzyl-trimethylammonium chloride, Alvéole, Paris) was added to cover the surface required. The sample was moved to the microscopy setup where the UV lithography was performed. The digital pattern was projected on top of the glass slide using UV light with a dose of 1,000 mJ/mm^2^ using PRIMO (Alvéole Lab). The samples were then washed three times using sterile PBS under a biosafety cabinet and incubated with fluorescently labeled (rhodamine) fibronectin (Tebu-bio, 10 μg/mL) for 5 min. Finally, the substrates were washed with EtOH for 10 min and then washed extensively with sterile PBS to remove excess rhodamine-fibronectin.

### Fluorescence staining and imaging

After the duration of the experiments (i.e. 2, 4, and 8 days), cells were fixed for 20 min at room temperature with 3.7% paraformaldehyde and after 10 min with Triton-X to let cytoplasmic proteins precipitate. Staining was performed using DAPI (D9542, Sigma–Aldrich), VCL (1:600 mouse anti-vinculin IgG1 antibody, Sigma, V9131), F-actin (Phalloidin-Atto647), FAP (1:600, rabbit polyclonal anti-FAP, Sigma–Aldrich), αSMA (1:600, IgG2A, mouse anti-αSMA, Sigma–Aldrich), and Atto 590 conjugated Phalloidin (Merck Life Science NV). The secondary antibodies used were for αSMA Alexa Fluor 488 conjugated (goat anti-mouse IgG2a, Molecular Probes), VCL Alexa Fluor 647 conjugated (goat, anti-mouse IgG1, Molecular Probes), FAP Alexa Fluor 647 conjugated (goat anti-rabbit, Molecular Probes). Fluorescence images were taken using a confocal laser scanning microscope (Leica SP8X equipped with 20×/0.75 or 40×/0.95 objective lens with a scanning speed of 100 Hz and the use of gating to increase the signal/noise ratio).

### Automated image analysis

The automated image analysis consists of three automatically run sequential steps: image preprocessing (using Python), morphology analysis (using CellProfiler), and data postprocessing (using Python; SI Appendix, Fig. S3). First, the image collection stored in native lif format was processed using a custom-written Jython python library. Single “.tiff” files were saved in separate folders where the images would be divided into single images per channel as well as the compressed stacks acquired per channel using the maximum projection function. The final step for the preprocessing of the input images was to change the image saturation to 0.35 by adjustment of the contrast and application of a Top Hat filter to the FAs channel images. The images were then used as inputs for the CellProfiler pipeline. First, the nuclei were identified through the IdentifyPrimaryObjects tool where the grayscale uploaded image was segmented using the Otsu two classes method with the possibility of manual filtering of the result using the EditObjectsManually tool. Cell boundaries were defined using IdentifySecondaryObjects and filtered as in the case of nuclei. The FAs, on the other hand, were identified through the previous masking of the cell boundaries and then converted into objects to be analyzed following a segmentation through the thresholding function. Quantitative information was obtained using tools like MeasureObjectsSizeShape and MeasureImageAreaOccupied. The output information was stored as.xlsx files. The analyzed data (in.xlsx) related to specific experimental conditions were processed using another custom-written Python library. The files were processed to take into account the desired parameters per object and stored as different.xlsx while generating summary graphs per object and graphs with the comparison between different experimental conditions.

The orientation of cytoskeleton components was carried out using FOAtool (37). The fluorescence channels of interest, actin and αSMA, were converted into .png. The .png files were imported in the MATLAB GUI and the orientation computed by using a constant combination of vesselness parameters.

### Atomic force microscopy

Cell cytoskeleton mechanics was determined using an atomic force microscope (Bruker Bioscope Catalyst mounted on a Leica DMi6000B inverted optical microscope). Measurements were performed with four-side pyramidal tips attached to cantilevers with a nominal spring constant of 0.01 N/m (MLCT-BIO, Bruker). Force–displacement curves were recorded at the perinuclear area of the cell at 1 Hz with a peak-to-peak amplitude of 3 μm. A minimum of 25 cells per condition was measured. The cells were kept outside the incubator for a maximum of 1 h, during the measurements. The Young's modulus was determined by fitting Hertz's model using NanoScope analysis software (Bruker) (49).

### Quantitative real-time PCR

Quantitative real-time PCR (qRT-PCR) was performed, as previously described (50). Briefly, total RNA from 1 × 10^5^ cells were isolated by the TRIzol RNA isolation protocol. QuantiTect RT kit (Qiagen) was then used to make cDNA from the RNA samples, following manufacturer's instructions. Relative gene expression was determined by qRT-PCR on a BioRad CFX384 real-time system using ABsolute QPCR SYBR Green mix (Thermo Fisher Scientific). Gene expression was determined by correcting for reference gene values (GAPDH), and the calculated values were expressed relative to the control group per experiment by means of the ΔΔC_t_ method. For a list of used primers, see SI Appendix, Table S1.

### Statistical analysis

Data are plotted as mean ± SD. Statistical analyses were performed using GraphPad Prism 9. One-way ANOVA was used to analyze the significance of comparing the experimental conditions to the control condition. Furthermore, we used Dunnett's multiple comparison test to compare the mean of each group with the control condition.

## Supplementary Material

pgae289_Supplementary_Data

## Data Availability

The data supporting the findings of this study are available at https://doi.org/10.5281/zenodo.12787416 and the automated image analysis pipeline is available at https://github.com/Kirkp95/CellProfilerDataAnalysis.
