# Supplementary material for: Spatial regulation of substrate adhesion directs fibroblast morphotype and phenotype
Source: PNAS Nexus. 2024 Jul 25;3(8):pgae289. doi: 10.1093/pnasnexus/pgae289 (PMC11316223; doi:10.1093/pnasnexus/pgae289)

**
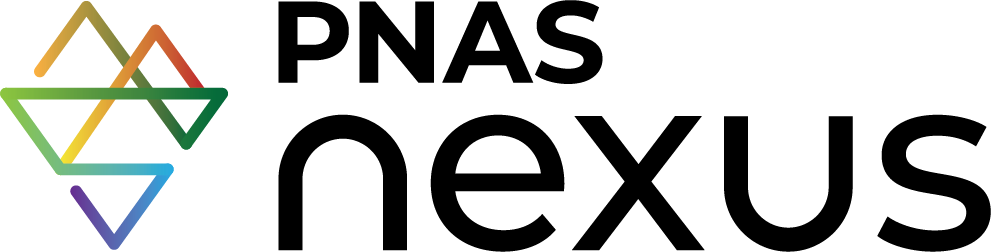
**

**Supplementary Information for**

Spatial regulation of substrate adhesion directs fibroblast morphotype and phenotype

Mirko D’Urso^1,2^, Ignasi Jorba^1,2,3^, Atze van der Pol^1,2^, Carlijn V.C. Bouten^1,2^, Nicholas A. Kurniawan* ^1,2^

^1^ Department of Biomedical Engineering, Eindhoven University of Technology, Eindhoven, The Netherlands

^2^ Institute for Complex Molecular Systems, Eindhoven University of Technology, Eindhoven, The Netherlands

^3^ Unitat de Biofísica i Bioenginyeria, Facultat de Medicina i Ciències de la Salut, Universitat de Barcelona, 08036 Barcelona, Spain

*Nicholas A. Kurniawan

Email: [n.a.kurniawan@tue.nl](mailto:n.a.kurniawan@tue.nl)

**This PDF file includes:**

Figures S1 to S11

Tables S1 to S2


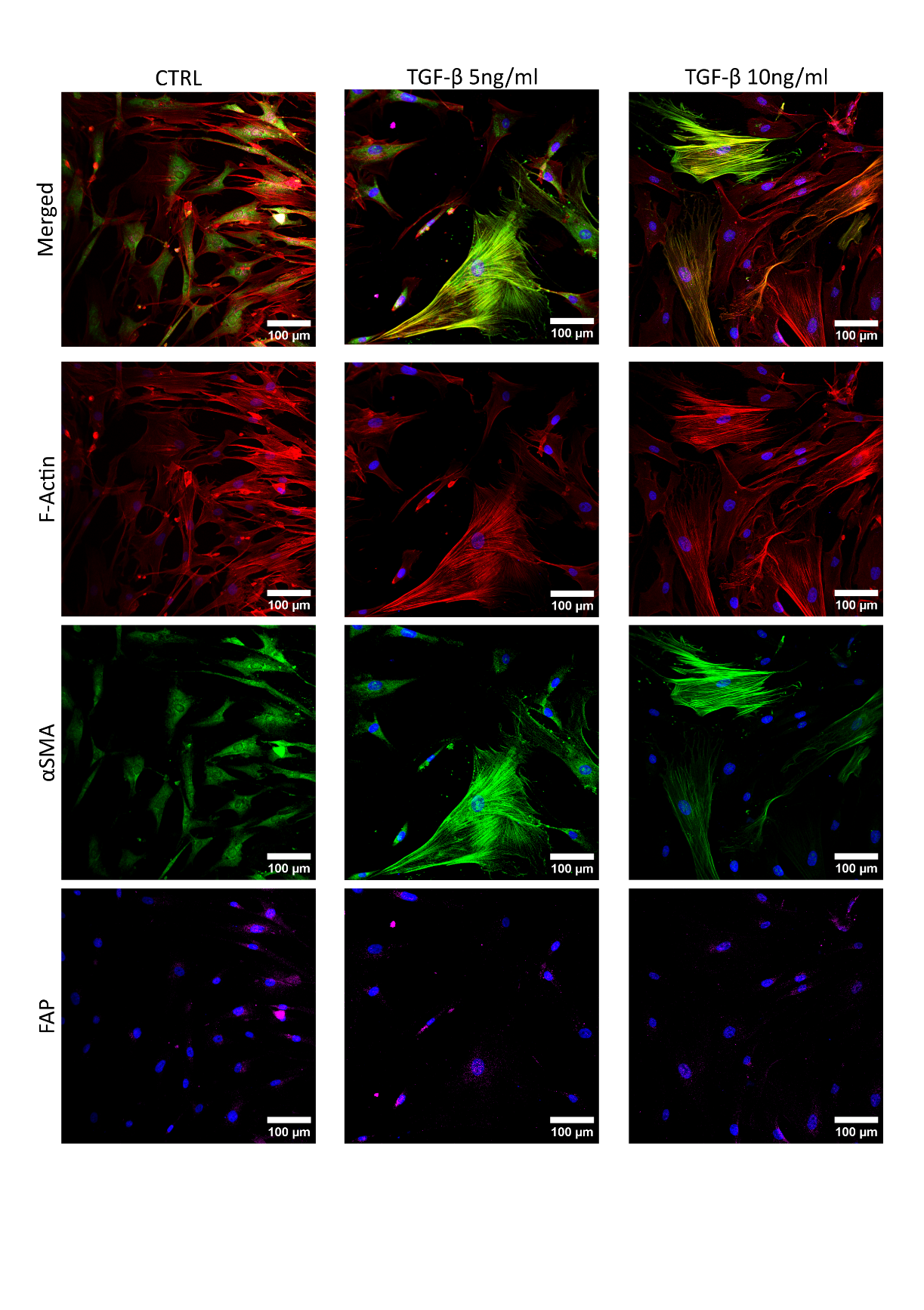


Fig. S1. Influence of TGF-β1 at different concentrations on fibroblast activation, showing the increase of αSMA expression and incorporation into actin stress fibers with increasing TGF-β1 concentration.


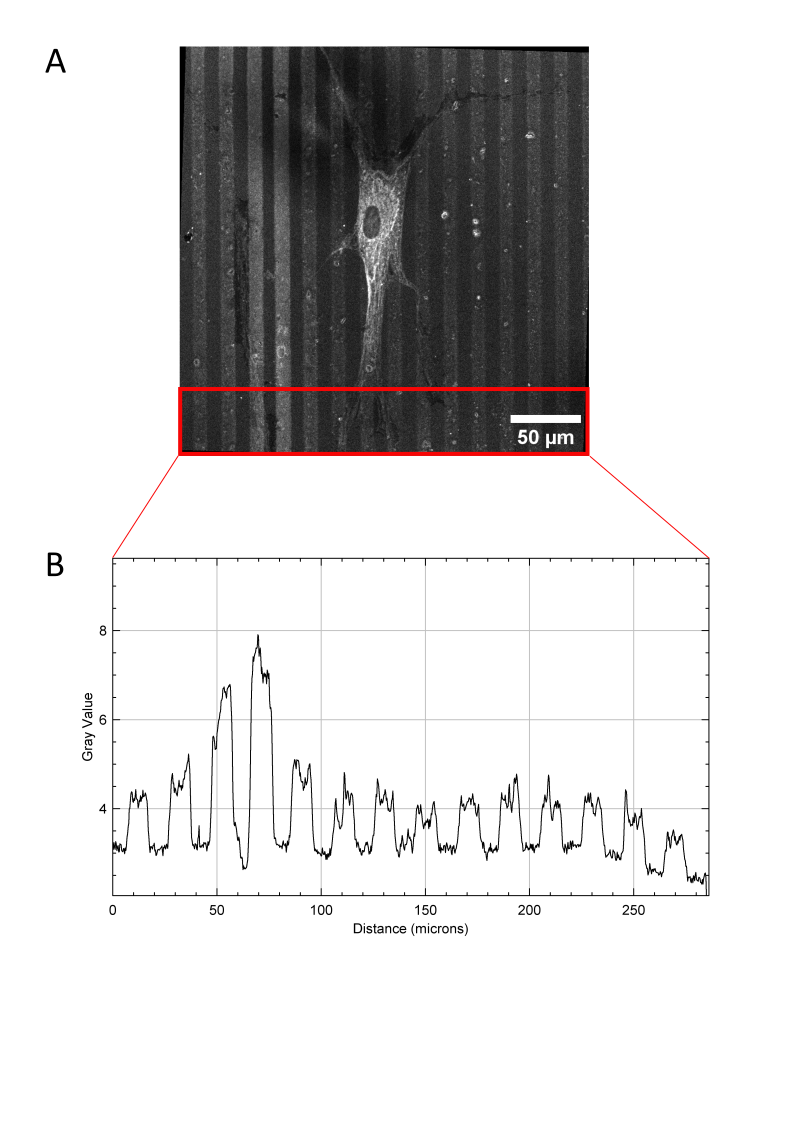


Fig. S2. A representative example of the stability of the protein micropatterns after 8 days of cell culture, as shown from the fluorescence image (A) and intensity profile of the fluorescently labeled fibronectin of the selected region (red box) (B).


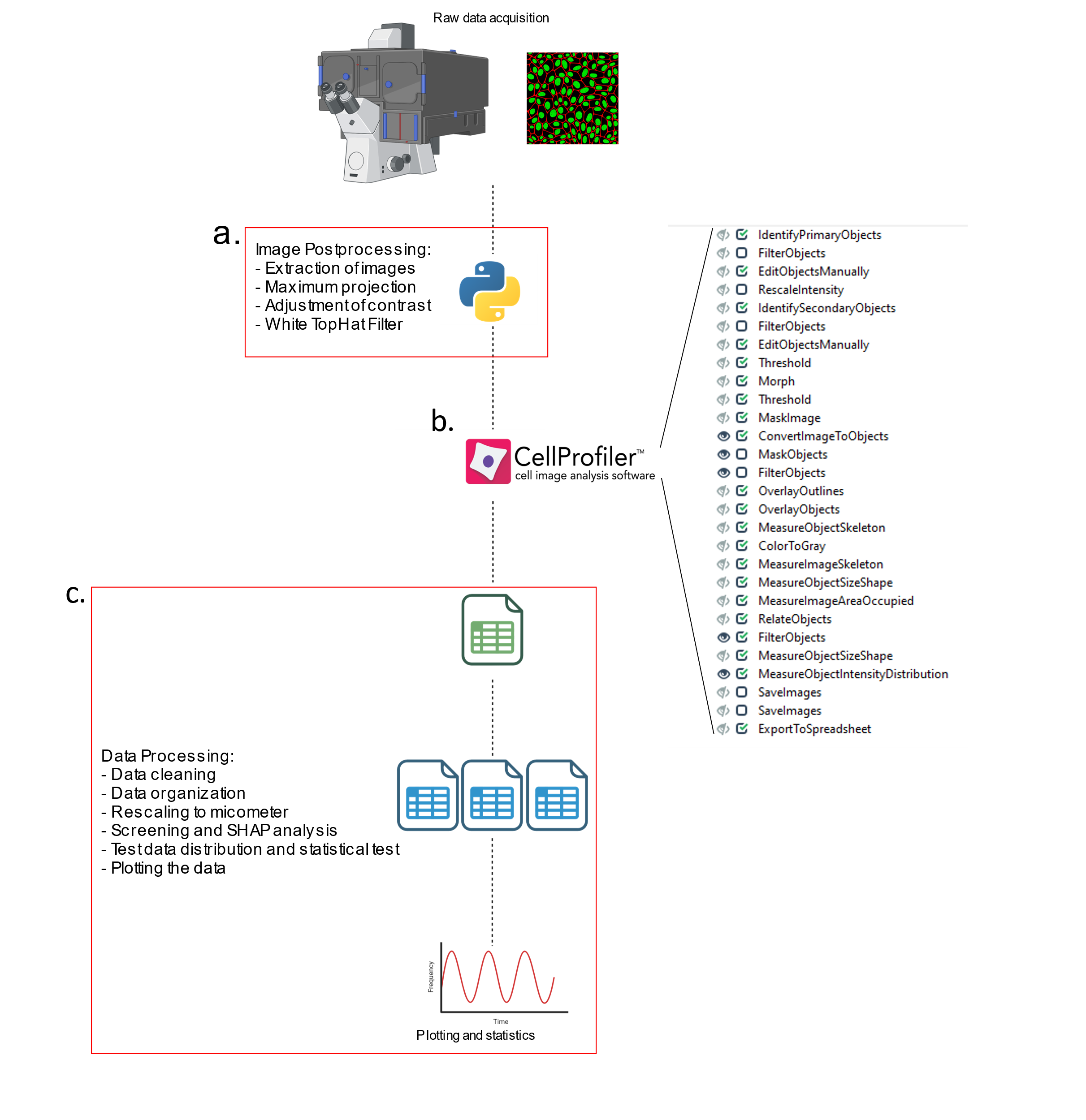


Fig. S3. Workflow of the morphometric image analysis. a) Handmade Python library has been designed to postprocess the raw images automatically through the use of customized ImageJ macros. b) Pipeline used in CellProfiler to process the postprocessed images. c) A second handmade custom Python library has been developed to process the CellProfiler output from a single Excel file to the production of plots and related statistical analysis.


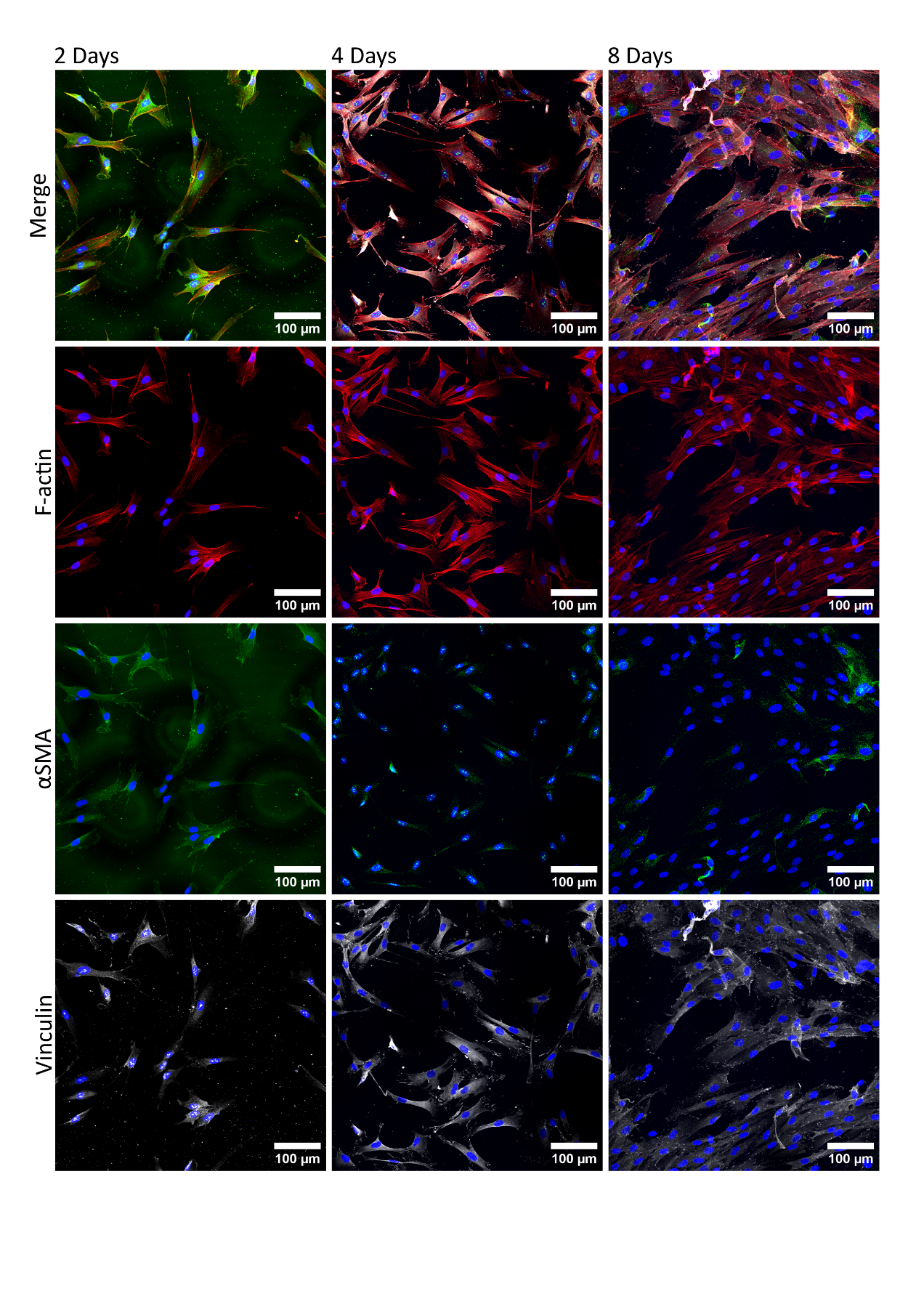
Fig. S4. Staining for DAPI (blue), αSMA (green), f-actin (red), and vinculin (gray) of fibroblasts on homogeneous coated fibronectin substrates at different time points.


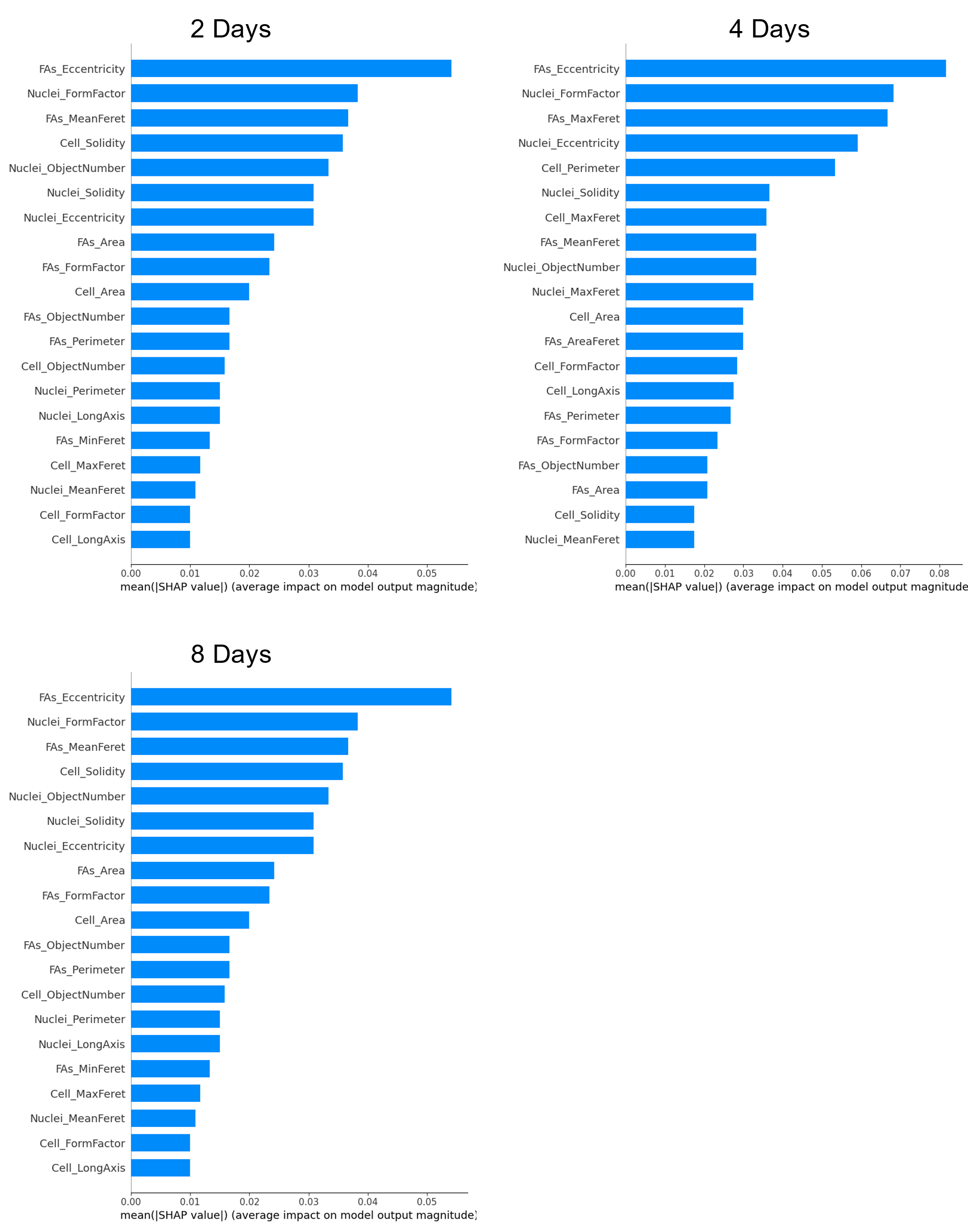


Fig. S5. SHAP values analysis carried out on different time points of fibroblast cultured on the micropatterns.


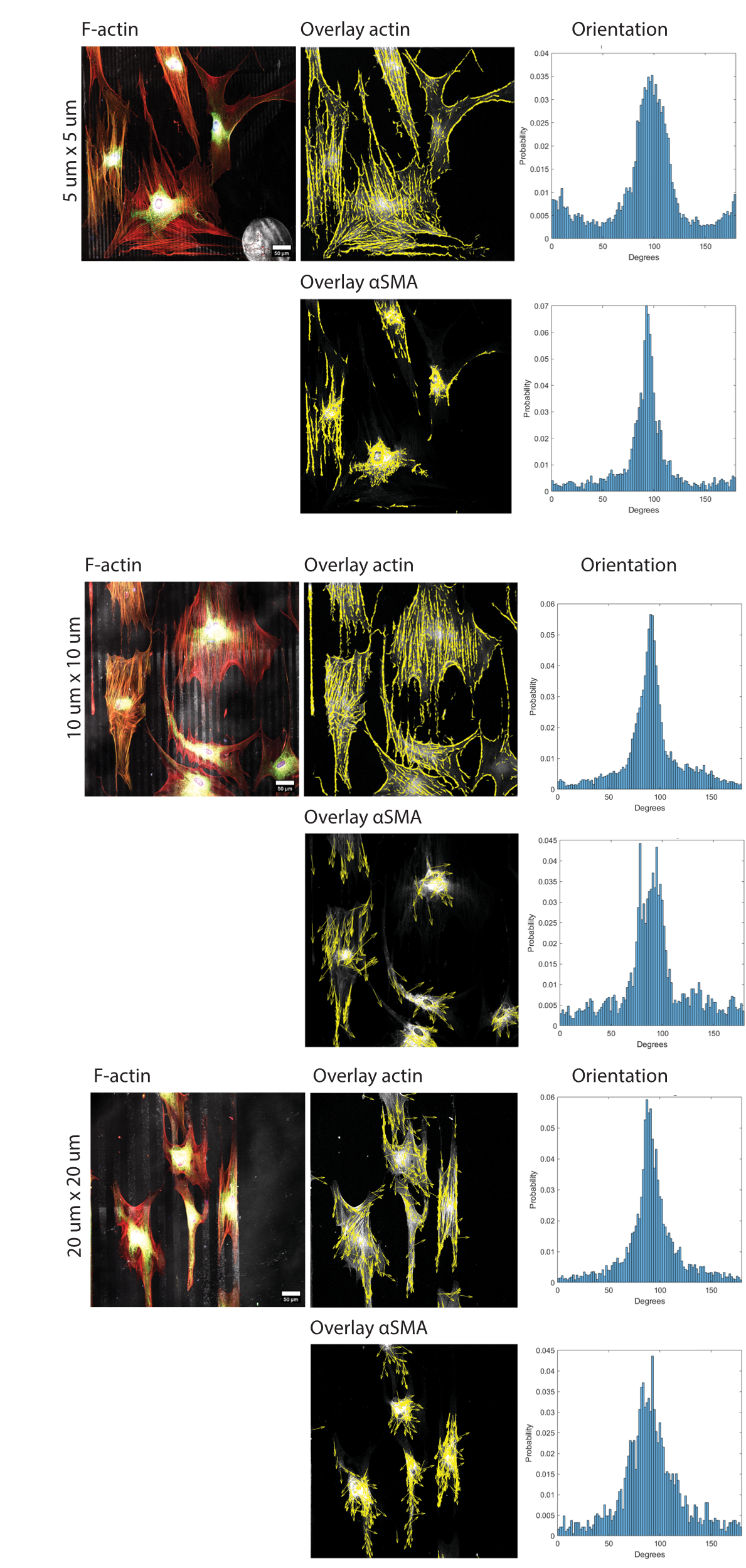


Fig. S6. Example of the orientation analysis performed by using FOAtool of the cytoskeleton components. The detected fibers (yellow) are overlaid to the corresponding raw fluorescence channels for αSMA and actin, and the orientation distribution of the detected fibers are plotted for each channel.


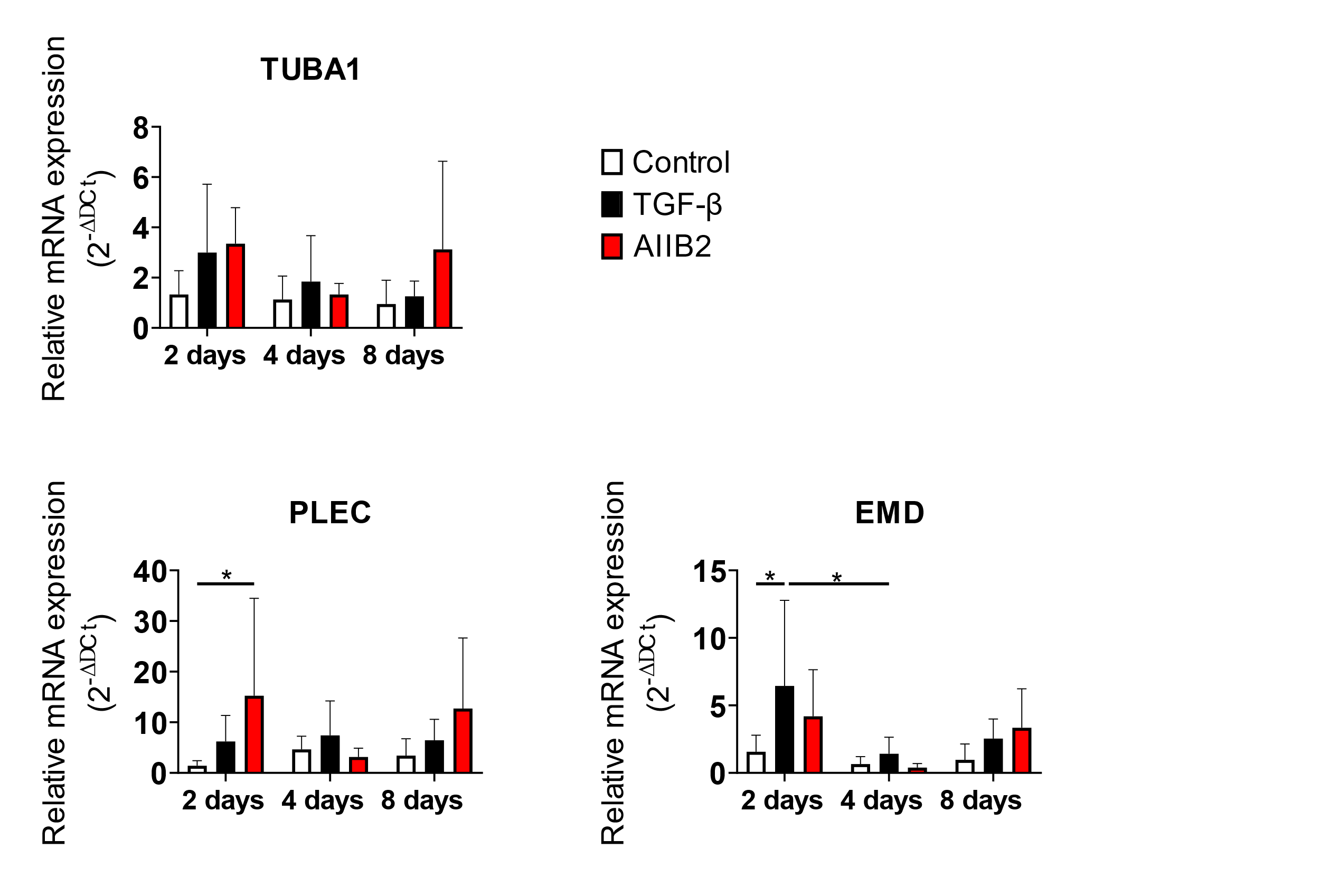


Fig. S7. qPCR result for fibroblasts on flat unpatterned condition for Tubulin1 (TUBA1), Plectin (PLEC), Emerin (EMD).


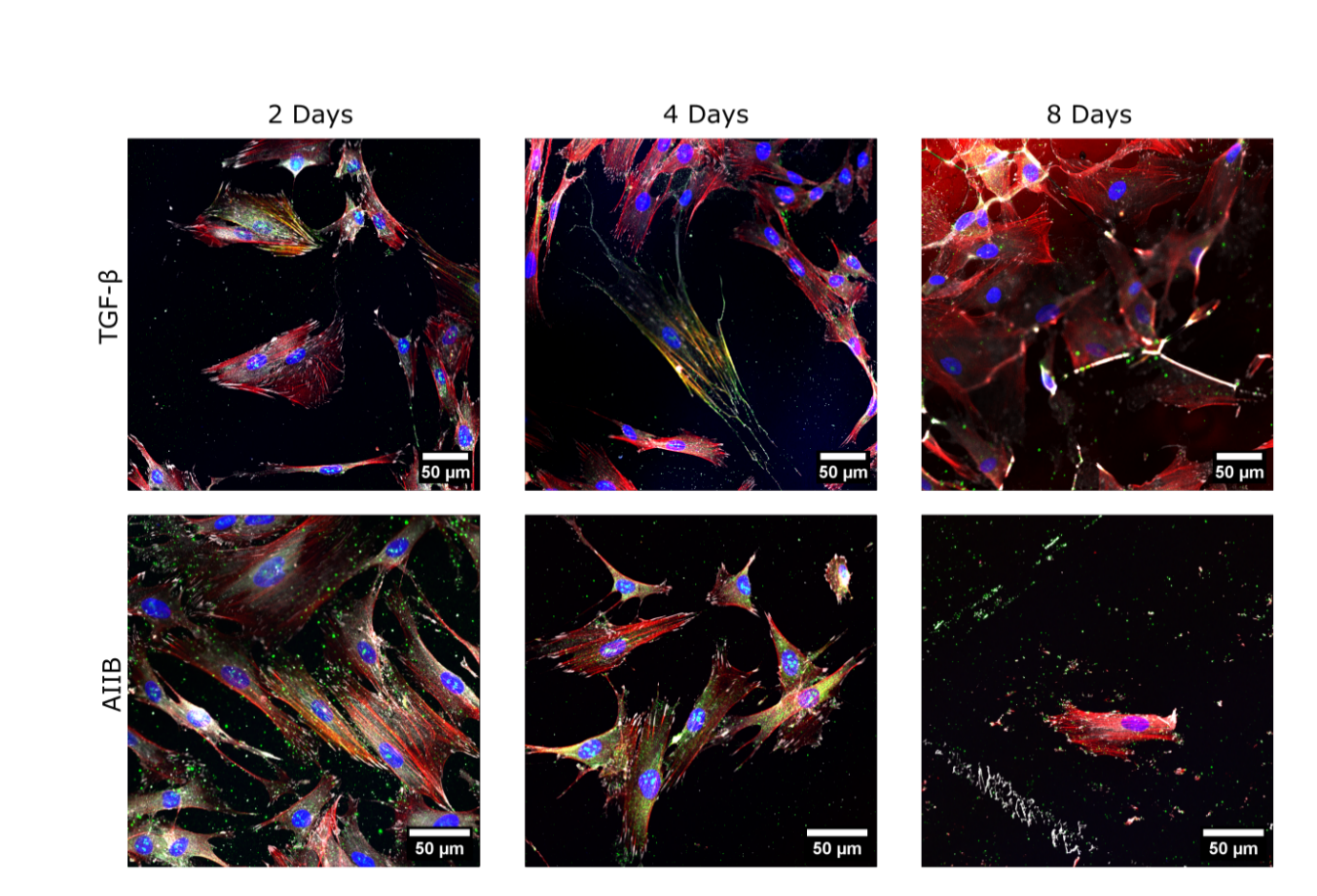


Fig. S8. Staining for DAPI (blue), αSMA (green), f-actin (red), and vinculin (gray). The addition of TGF-β increases the activated fibroblasts (top row) by the formation of αSMA, while the addition of integrin β2 inhibitor prevents fibroblast activation (bottom row).


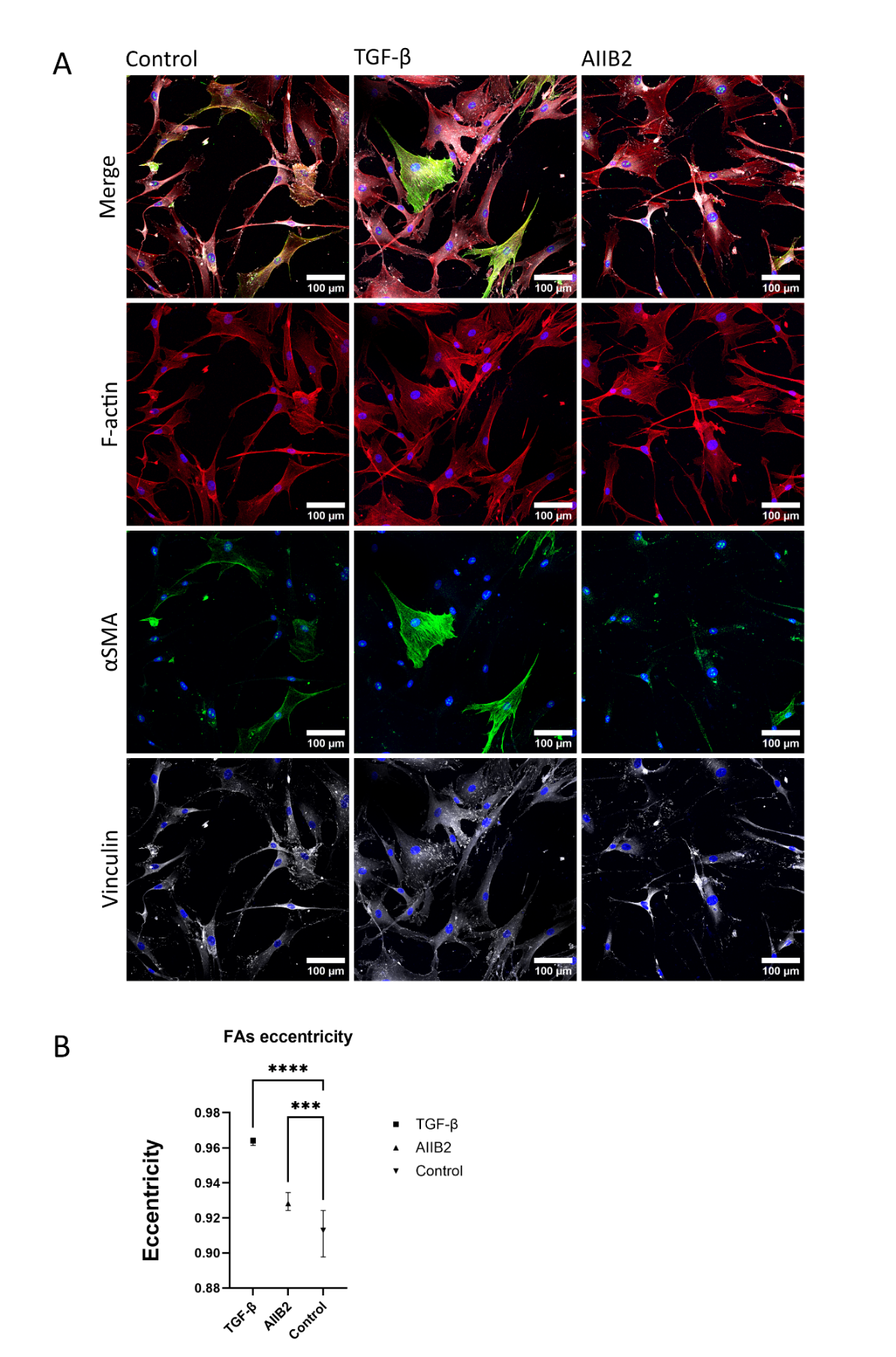


Fig. S9. A) Staining for DAPI (blue), αSMA (green), f-actin (red), and vinculin (gray) on homogeneous fibronectin-coated substrate. Fibroblasts were cultured for 4 days with the addition of TGF-β, which triggers their activation, as indicated by αSMA stress fibers, while the addition of integrin β2 inhibitor prevents it. B) Focal adhesion eccentricity of cells under these three conditions. *** p<0.0005 , **** p<0.00005 (Kruskal-Wallis test).


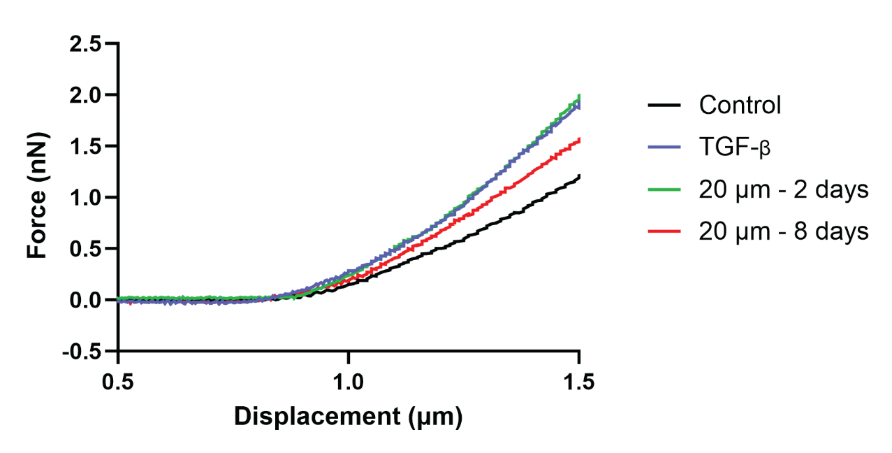


Fig. S10. Representative force–indentation curves for different fibroblast culture conditions, from which the cell’s Young’s modulus was obtained.


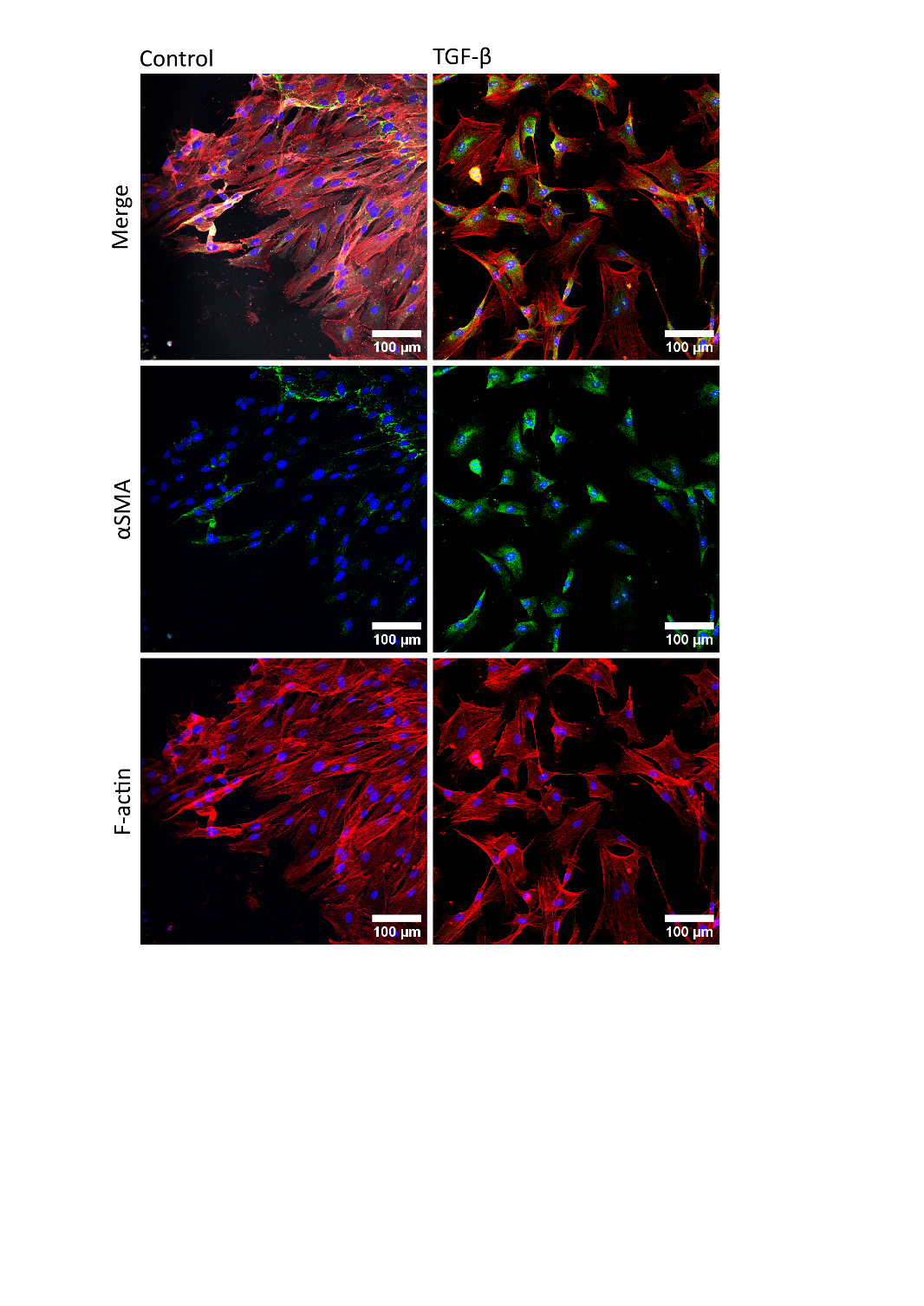


Fig. S11. Staining for DAPI (blue), αSMA (green), f-actin (red) of fibroblasts cultured for 8 day with and without 10 ng/ml TGF-β. The induced fibroblasts present a higher amount of cytoplasmatic αSMA albeit the cellular morphology is similar to myofibroblast.

Table S1. Primers used for qPCR.

| Gene Name | Symbol | Primer | Sequence 5' to 3' |
| --- | --- | --- | --- |
| Glyceraldehyde-3-Phosphate Dehydrogenase | *GAPDH* | Forward | GGTGAAGGTCGGAGTCAACG |
|  |  | Reverse | GCTTCCCGTTCTCAGCCTT |
|  |  |  |  |
| Vinculin | *VCL* | Forward | ACCTTGAACAACTCCGACTAAC |
|  |  | Reverse | AACTCTTCATCCTTTTCCTCTGG |
|  |  |  |  |
| Vimentin | *VIM* | Forward | CAAGACCTGCTCAATGTTAAGATC |
|  |  | Reverse | CTGCTCTCCTCGCCTTCC |
|  |  |  |  |
| Plectin | *PLEC* | Forward | GCCAGAGGTTTGCGAAACAG |
|  |  | Reverse | TTGTACGTCACCAGCTGGAG |
|  |  |  |  |
| Emerin | *EMD* | Forward | TCACCAGGTGCATGATGACG |
|  |  | Reverse | GGCGTTCCCTATCCTTGCA |
|  |  |  |  |
| Tubulin Alpha Chain | *TUBA1* | Forward | GAGTGCATCTCCATCCACGT |
|  |  | Reverse | CCGTGTTCCAGGCAGTAGAG |
|  |  |  |  |
| Smooth Muscle Actin | *αSMA* | Forward | CGTGTTGCCCCTGAAGAGCAT |
|  |  | Reverse | ACCGCCTGGATAGCCACATACA |
|  |  |  |  |
| Protein Tyrosine Kinase 2 | *PTK2* | Forward | TGACGGCACCATCCCTAAC |
|  |  | Reverse | GACTGCGAGGTTCCATTCAC |
|  |  |  |  |

Table S2. List of parameters quantified using morphometric image analysis


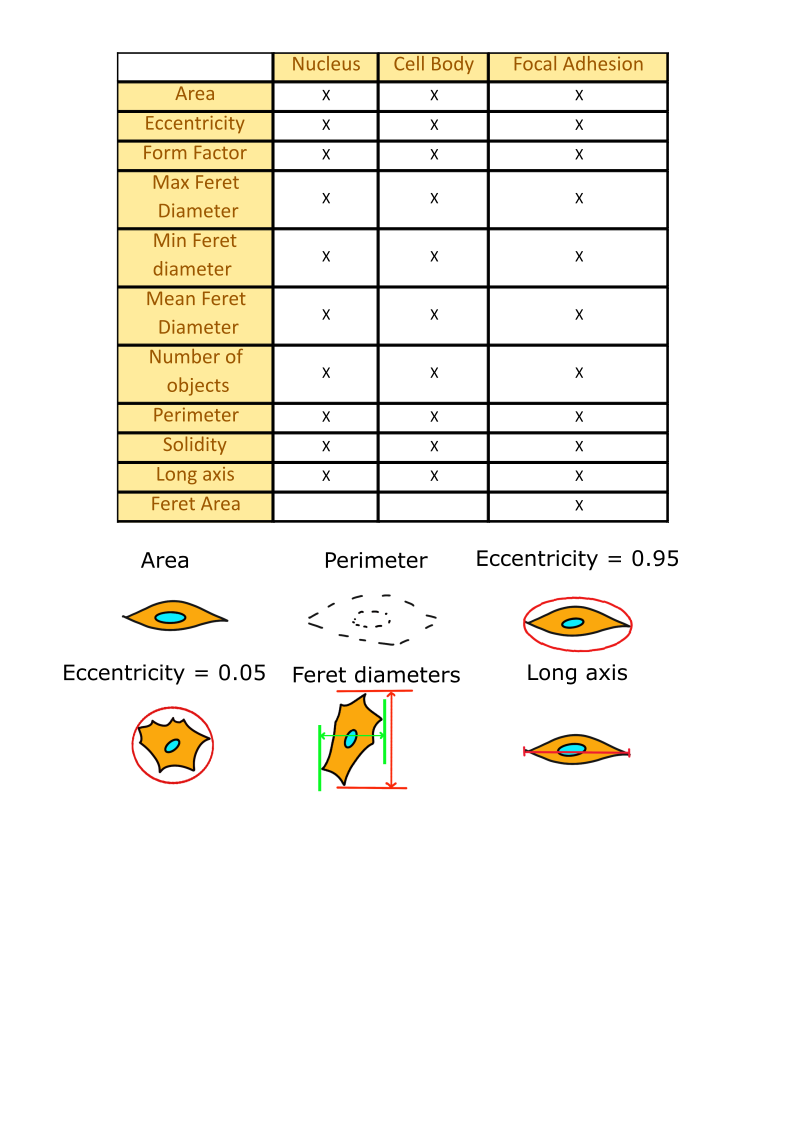

Supplement: pgae289_Supplementary_Data [file pgae289_supplementary_data.docx]
